# Neutrophil Extracellular Traps Directly Induce Epithelial and Endothelial Cell Death: A Predominant Role of Histones

**DOI:** 10.1371/journal.pone.0032366

**Published:** 2012-02-28

**Authors:** Mona Saffarzadeh, Christiane Juenemann, Markus A. Queisser, Guenter Lochnit, Guillermo Barreto, Sebastian P. Galuska, Juergen Lohmeyer, Klaus T. Preissner

**Affiliations:** 1 School of Medicine, Institute of Biochemistry, Justus-Liebig-University, Giessen, Germany; 2 Pulmonary and Critical Care Medicine, Northwestern University, Chicago, Illinois, United States of America; 3 Max-Planck-Institute for Heart and Lung Research, Bad Nauheim, Germany; 4 Department of Internal Medicine II, Justus-Liebig-University, Giessen, Germany; University of Tübingen, Germany

## Abstract

Neutrophils play an important role in innate immunity by defending the host organism against invading microorganisms. Antimicrobial activity of neutrophils is mediated by release of antimicrobial peptides, phagocytosis as well as formation of neutrophil extracellular traps (NET). These structures are composed of DNA, histones and granular proteins such as neutrophil elastase and myeloperoxidase. This study focused on the influence of NET on the host cell functions, particularly on human alveolar epithelial cells as the major cells responsible for gas exchange in the lung. Upon direct interaction with epithelial and endothelial cells, NET induced cytotoxic effects in a dose-dependent manner, and digestion of DNA in NET did not change NET-mediated cytotoxicity. Pre-incubation of NET with antibodies against histones, with polysialic acid or with myeloperoxidase inhibitor but not with elastase inhibitor reduced NET-mediated cytotoxicity, suggesting that histones and myeloperoxidase are responsible for NET-mediated cytotoxicity. Although activated protein C (APC) did decrease the histone-induced cytotoxicity in a purified system, it did not change NET-induced cytotoxicity, indicating that histone-dependent cytotoxicity of NET is protected against APC degradation. Moreover, in LPS-induced acute lung injury mouse model, NET formation was documented in the lung tissue as well as in the bronchoalveolar lavage fluid. These data reveal the important role of protein components in NET, particularly histones, which may lead to host cell cytotoxicity and may be involved in lung tissue destruction.

## Introduction

Neutrophils are the most abundant type of white blood cells in mammals, which represent an essential part of the innate immune system and are considered as the first line of defense against microorganisms. After recruitment to the inflammatory site, neutrophils attack invading pathogens by release of antimicrobial peptides and lytic enzymes as well as production of reactive oxygen species (ROS) followed by phagocytosis that enables clearance of the invading pathogens [1]–[3]. Another recently described antimicrobial mechanism of neutrophils is the formation of neutrophil extracellular traps (NET). These structures are composed of DNA in association with histones, as the most abundant proteins in NET, as well as granular proteins such as elastase and myeloperoxidase and several cytoplasmic proteins. Inflammatory stimuli such as interleukin-8, lipopolysaccharide (LPS) or phorbol myristate acetate (PMA) provoke “NETosis” of neutrophils. During this cell-destructive process, which is distinct from classical apoptosis or necrosis, intracellular organelle membranes disintegrate after decondensation of materials in the nucleus, allowing the mixing of cytoplasmic and nuclear components which is followed by the rupture of plasma membrane to expel NET. These structures can bind and kill bacteria and fungi [4]–[7], whereby NET-associated proteins such as elastase and histones exhibit bactericidal and leishmanicidal activity [5], [8].

However, excessive activation of neutrophils may lead to the development of multiple organ dysfunction syndrome, and lungs are the main target of this syndrome [9]–[11]. Acute lung injury (ALI) and its more severe form acute respiratory distress syndrome (ARDS) represent pathological situations of lung dysfunction characterized by impairment in the alveolar-capillary barrier function that result from complex responses of the lung to a multitude of direct and indirect insults [12]–[14]. Activated neutrophils contribute to lung injury by releasing proteolytic enzymes, ROS and other proinflammatory mediators [15]. Alveolar epithelial cell function and barrier integrity are crucial to preserve normal gas exchange, and injury or loss of epithelial cells may lead to progression of ALI/ARDS [16], [17]. ARDS carries high mortality rates between 40% and 60% in affected patients [18]. Therefore, understanding of the mechanisms in the development of ALI/ARDS is essential for developing novel therapeutic options to treat these patients [19], [20]. Besides ALI, other lung diseases such as cystic fibrosis are associated with lung epithelial cell death. In cystic fibrosis patients, progressive infection and inflammation in the lower airways results in the destruction of small and medium airways in lung, and extracellular DNA accumulates in the airway due to the chronic bacterial infection [21].

Although elevated amounts of NET were observed in several pathophysiological conditions *in vivo* [5], [22]–[28] and exaggerated NET formation was correlated with damaging effects and impaired tissue function [29]–[31], the direct effect of NET and their components on host cells have not been investigated. In this study, the direct influence of isolated NET on alveolar epithelial cells (as the major cells responsible for gas exchange in the lung) as well as endothelial cells was examined, indicating that NET and particularly the histone components are responsible for cell death in lung epithelial and endothelial cells.

## Materials and Methods

### Cell culture

A549-human lung adenocarcinoma cell line and mouse lung epithelial cells (MLE-12) were obtained from American Type Culture Collection (ATCC, Manassas, VA, USA). Human pulmonary artery endothelial cells (HPAEC) were obtained from Lonza (Germany). Human umbilical vein endothelial cells (HUVEC) and murine alveolar type II (AT-II) cells were isolated as described [32], [33]. Human neutrophils were isolated from healthy donors using density gradient separation according to Costa and co-workers [34]. Briefly, a double gradient was formed by layering an equal volume of histopaque-1077 over histopaque-1119 (Sigma-Aldrich, Germany). Venous blood was collected in EDTA tubes and carefully layered onto the upper histopaque-1077, followed by centrifugation at 700× g for 30 min and granulocytes were concentrated at the 1077/1119 interphase. Purity of isolated cells (>96%) was assessed by FACS following labeling the cells with neutrophil-specific marker CD66b (antibodies-online, Germany), and viability was determined to be 98% by trypan blue dye exclusion. For neutrophil isolation, human blood taken from healthy volunteer donors with verbal consent was provided by the Blood Bank (Clinical Immunology, Blood Transfusion Medicine, University Hospital Giessen) and data were analyzed anonymously.

### Isolation of neutrophils from bronchoalveolar lavage fluid (BALF) and immunofluorescence microscopy of isolated neutrophils

To generate ALI, C57BL/6 mice (n = 3 per group) were treated intratracheally with 10 µg of LPS from *Escherichia coli* (serotype 0111.B4, Sigma Aldrich) in 50 µl of PBS. Control mice were treated with PBS only [35]–[37]. Intratracheal applications of LPS or PBS were done essentially as described elsewhere [38], [39]. Briefly, mice were anesthetized with ketamine and tetrazoline hydrochloride, and the trachea was exposed. Subsequently, catheter (Abbot, Wiesbaden, Germany) was inserted into trachea, and LPS or PBS was installed under stereomicroscopic control (MS5; Leica Microsystems, Wetzlar, Germany). After installation, wounds were closed, and mice were allowed to recover with free access to food and water. For BALF collection, mice were killed with an overdose of isoflurane (Forene; Abbott, Wiesbaden, Germany). Subsequently, trachea was exposed, and a small incision was made to insert a shortened 21-gauge cannula connected to a 1-ml insulin syringe, followed by repeated intratracheal instillations of 0.5 ml aliquots of PBS [38], [39]. After 24 h, BALF was collected and mouse neutrophils were isolated by anti-Ly-6G MicroBead Kit (Miltenyi Biotec, Germany) according to the manufacturer's protocol. Isolated neutrophils were seeded on coverslips and treated with 25 nM PMA (Sigma-Aldrich) for 1.5 h at 37°C. Samples were fixed with 2% paraformaldehyde, blocked with 3% bovine serum albumin in PBS and incubated with primary mouse anti-DNA Histone H1 (Millipore, Germany) and rabbit anti-CD46 (H-294) (Santa Cruz, Germany), followed by detection with secondary antibodies coupled to Alexa Fluor 555 donkey anti-mouse IgG and Alexa Fluor 488 donkey anti-rabbit (Invitrogen, Germany), respectively. DAPI (Vectashield mounting medium with DAPI; Vector Laboratories, Burlingame, CA, USA) was used for nuclear DNA detection. For negative controls either the primary antibodies were omitted or the isotype-matched controls were used. Images were taken with fluorescence microscope using MetaMorph imaging software version series 7.0 (Leica Microsystems, Wetzlar, Germany). All animal experiments were approved by ethical approval (approval ID 67/2009) from the Ethics Committee of the University of Giessen, School of Medicine.

### BALF collection, MNase digestion and neutrophil elastase activity

C57BL/6 mice were treated intratracheally with LPS as mentioned above, and BALF was collected after 3, 6, 12, 24 and 48 h following treatment. Control mice were treated with PBS only. BALF samples were centrifuged 1000× g for 5 min. The supernatants were collected separately and 500 mU/ml micrococcal nuclease from *Staphylococcus aureus* (MNase) (Sigma-Aldrich) were added to each pellet [4]. Both digested pellet (to detect NET-derived elastase) and supernatant (to detect free elastase) were incubated with peptide substrate N-(methoxysuccinyl)-Ala-Ala-Pro-Val 4-nitroanilide (Sigma Aldrich) for 15 min and the optical density was measured at 405 nm (Ultra microplate reader ELx 808; BIO-TEK Instruments, Germany).

### Immunofluorescence microscopy of mouse lung

Frozen 5 µm lung tissue sections from mice treated intratracheally for 24 h with 10 µg LPS or PBS only were equilibrated to room temperature and fixed in acetone for 10 min. Vector M.O.M. Immunodetection Kit (Vector Laboratories) was used with some modification in the protocol. Briefly, after blocking the sections with M.O.M Mouse Ig Blocking Reagent for 1 h, samples were incubated with primary antibodies anti-CD46 or anti-neutrophil elastase (M-18; Santa Cruz) followed by a secondary antibody coupled to Alexa Fluor 488 and anti-DNA/histone 1 coupled with Alexa Fluor 546 (Invitrogen). In addition, some sections were incubated with primary antibodies against myeloperoxidase (L-20; Santa Cruz) and citrullinated H3 (2+8+17) [CitH3] (Abcam), followed by detection with secondary antibodies coupled to Alexa Fluor 555 donkey anti-mouse IgG and Alexa Fluor 488 donkey anti-rabbit, respectively. For negative controls either the primary antibodies were omitted or the isotype-matched controls were used. DAPI was used for DNA detection.

### Histone treatment of cells

A549 cells and HUVEC were treated with different concentrations of histone type IIA from calf (Sigma-Aldrich). Cell morphology was inspected under the light microscope and the cell numbers were counted by CASY Cell Counter System (Schaerfe Systems, Reutlingen, Germany).

### NET production, isolation and quantification

Isolated human neutrophils were resuspended in phenol red–free RPMI 1640 (Invitrogen), and 1.8×10^6^ cells were seeded per well in 6-well plates. Following stimulation with 50 nM PMA for 4 h, medium was removed and wells were washed with RPMI. PMA, at this concentration, does not promote apoptosis or necrosis; rather, it induces typical features of NETosis [4], [40]–[44]. To collect NET, 2 ml RPMI per well was added and NET (the smear on the wells) was collected in 15 ml tubes by vigorous agitation. After centrifugation at 20× g for 5 min, NET was collected in the supernatant and subjected to different treatments, including partial digestion by 500 U/ml MNase for 10 min at 37°C or complete digestion by 10 U/ml DNase I (Fermentas, Germany) for 20 min at 37°C or kept undigested. For some experiments, NET was heated for 5 min at 95°C (“boiled NET”). Quantification of DNA in NET was performed by Picogreen dsDNA kit (Invitrogen) according to the manufacturer's instructions.

### Treatment of endothelial or epithelial cells with NET

HUVEC, HPAEC or A549, MLE-12, AT-II cells were seeded in 24-well plates to reach 90% confluency, washed once with PBS, and NET in different concentrations were added. Approximately 3.4 (NET) or 10.1 (3×NET) µg/ml DNA-NET, respectively, was added to different wells. Some cells were treated with 3.3 µg/ml staurosporine (Sigma-Aldrich) as a cell death inducer and some left untreated as negative controls. The total volume in each well was kept equal by adding RPMI medium. Cell morphology evaluation was also performed with the non-confluent cells by MetaMorph software using integrated morphometry analysis. Cells were analyzed after 4 h or 16 h incubation at 37°C with different measurements as indicated in the following methods.

### Lactate dehydrogenase (LDH) release or cytotoxcicity assay

Epithelial or endothelial cells were treated with 1% Triton X-100 (high control), with NET or kept untreated (low control). LDH release into the supernatant was assessed by cytotoxicity detection kit (Roche Applied Science, Germany) according to the manufacturer's instructions. The degree of cytotoxicity was calculated as follows:
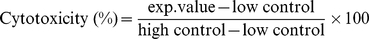
Where “exp.value” is the average absorbance (of four wells) from the experimental data.

### Multi-caspase activity

A549 cells were left untreated or treated with NET, staurosporine or caspase inhibitor Z-VAD-FMK as an additional negative control, and green multi-caspase staining kit (Promokine, Germany) was used to detect activated caspases in living cells according to the manufacturer's protocol. The fluorescence intensity was measured at exitation and emission wavelengths of 485 nm and 535 nm, respectively (FLx 800 fluorescence microplate reader; BIO-TEK Instruments).

### Detection of Annexin V and ethidium homodimer III positive cells by fluorescence microscopy

Untreated (control) and NET- or staurosporine-treated A549 cells were stained with FITC-Annexin V, ethidium homodimer III and Hoechst 33342 (Promokine, Germany) according to the manufacturer's instructions. Annexin V and ethidium homodimer III positive cells were evaluated by fluorescence microscopy and MetaMorph software using integrated morphometry analysis.

### Treatment of histone and NET by activated protein C (APC)

Histone from calf thymus (Type II-A; Sigma-Aldrich) (100 or 200 µg/ml) was incubated with 6 µg/ml (100 nM) APC (Xigris, Eli Lilly) for 1 h at 37°C [45]. In addition, NET (approximately 10 µg/ml protein content), digested or non-digested, was incubated with 100 nM APC for different time points. Moreover, NET was incubated with APC at different mass ratios of APC: NET (1∶5, 1∶2, and 1∶1) for 1 h at 37°C. APC alone or APC plus 6 µM APC inhibitor, PPACK (Calbiochem, Germany) were used as controls. LDH release by A549 cells was measured after treatment with histones, APC, NET, APC-pretreated histones or APC-pretreated NET.

### Elastase activity of human neutrophils and isolated NET

In order to evaluate elastase activity and its inhibition in NET, human neutrophils were kept untreated as unstimulated samples (Unstim) or stimulated with 50 nM PMA for 4 h (Stim) and the supernatants were collected for elastase activity. NET was also isolated from the stimulated samples as described under “NET production, isolation and quantification”. Thereafter, NET samples were digested with DNase or MNase or kept undigested followed by centrifugation at 1000× g for 5 min, and the supernatants were collected for elastase activity in the absence or presence of 0.2 mM N-(methoxysuccinyl)-L-alanyl-L-alanyl-L-prolyl-L-valine chloromethyl ketone (Sigma-Aldrich) as an elastase inhibitor.

### NET-protein quantification

For NET-protein quantification the Micro-BCA protein assay reagent kit (Pierce, Germany) or the 2D-Quant kit (GE Healthcare) was used. Both methods resulted in the same values of protein concentration.

### Inhibition of NET cytotoxicity

To test the influence of NET on cytotoxicity, 800 µl NET (approximately 10 µg protein content) was pre-incubated with 1∶100 of the following histone antibodies: H2A antibody (Cell signaling, Germany), H3 antibody (Millipore), citrullinated H3 (2+8+17) antibody [CitH3] (Abcam, Germany) or H4 antibody (Cell signaling) or with 8 µg of the subsequent antibodies: DNA/histone H1 antibody (Millipore, German), H2B antibody (Millipore), mouse IgG isotype antibody (M4509; Sigma-Aldrich) or rabbit IgG isotype antibody (Dianova, Germany) for 1 h at room temperature before incubation of NET with A549 cells. In addition, 800 µl NET was pre-incubated with 0.125, 0.25, 0.5 or 1 mM neutrophil elastase inhibitor N-(methoxysuccinyl)-Ala-Ala-Pro-Val-chloromethyl ketone, with 37 ng/ml myeloperoxidase inhibitor dihydrolipoic acid (gift from Dr. Oliver Soehnlein, Ludwig-Maximilians-University Munich) or with 10 ìg polysialic acid (colominic acid á2,8-linked Neu5Ac polymer of E.coli K1 - equivalent to polySia in mammalians) (Sigma-Aldrich) for 1 h at room temperature prior to incubation of NET with A549 or AT-II cells. As a control, 50 µg/ml isolated histones were pre-incubated with 50 µg/ml polysialic acid or 1∶100 antibody against histone H4 for 1 h at room temperature before incubation with the cells.

### Statistical analysis

Data were analyzed by GraphPad Prism 5.02 software using one-way analysis of variance (ANOVA) with Tukey post-tests for multiple comparisons or by student's t-test for single measurements. Each experiment was performed at least three times on independent occasions unless otherwise stated. Differences were considered statistically significant at *p*<0.05. In the figures, significant differences were illustrated with asterisks (*p<0.05; **p<0.01; ***p<0.001).

## Results

### NET induce cell death in epithelial cells in a concentration-dependent manner

**Figure 1:**
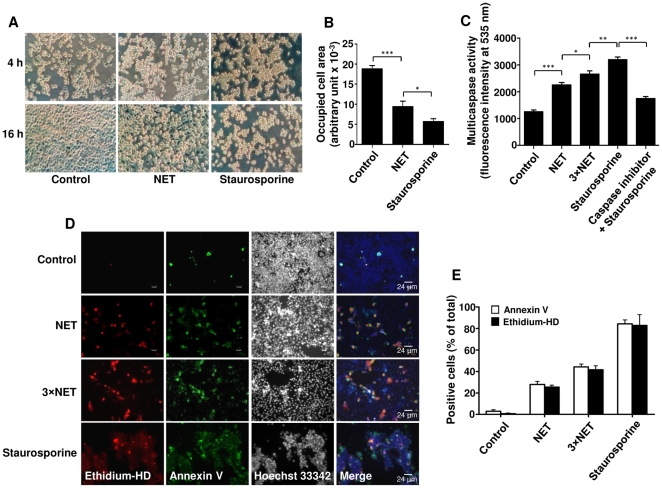
NET cause lung epithelial cell death in a concentration-dependent manner. (A) The morphology of A549 cells was evaluated after 4 or 16 h treatment with medium (control), NET or staurosporine. Shown are representative pictures of >8 independent experiments at 20× magnification. (B) Cell growth from (A) was quantified by measuring the difference between occupied cell area after 16 h and 4 h. (C) Multicaspase activity of A549 cells was measured after 16 h treatment with two concentrations of NET (3.4 and 10.1 µg/ml DNA-NET) or staurosporine. Shown are representative data of three independent experiments (mean 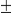 SD), *p<0.05; **p<0.01; ***p<0.001. (D) Immunofluorescence staining of A549 cells after 16 h treatment with two concentrations of NET (3.4 and 10.1 µg/ml DNA-NET) or staurosporine was performed for ethidium homodimer III (ethidium-HD, red), annexin V (green), and Hoechst 33342 (black and white). Shown are representative pictures of three independent experiments. (E) Percentage of ethidium-HD and annexin-V positive cells from (D) was evaluated by morphometry analysis.

To study the effect of NET on epithelial cells, A549 cells were incubated with MNase-digested NET or staurosporine for 4 h or 16 h. While untreated cells grew normally and became confluent, cells treated with staurosporine or NET did not reach confluency (Fig. 1A and B). Vigorous agitation of unstimulated neutrophils incubated for 4 h at 37°C will disturb the intact neutrophils, and may result in the release of all components of neutrophils which are not equivalent to NET, and cannot be used as controls; therefore, only the untreated cells were used as controls. Multicaspase activity in A549 cells increased significantly after exposure to staurosporine or NET in a dose-dependent manner (Fig. 1C). Moreover, NET exposure increased the fractions of annexin V and ethidium homodimer (a membrane-impermeable fluorescent dye which binds to DNA) positive cells (Fig. 1D and E). Proteomic analysis of NET-treated epithelial cells (Materials and Methods S1) also revealed up-regulation of proteins involved in cell death (Table S1). Together, these data indicate that NET can induce lung epithelial cell death in a concentration-dependent manner.

### NET induce cytotoxicity in epithelial and endothelial cells independent of DNA digestion

**Figure 2:**
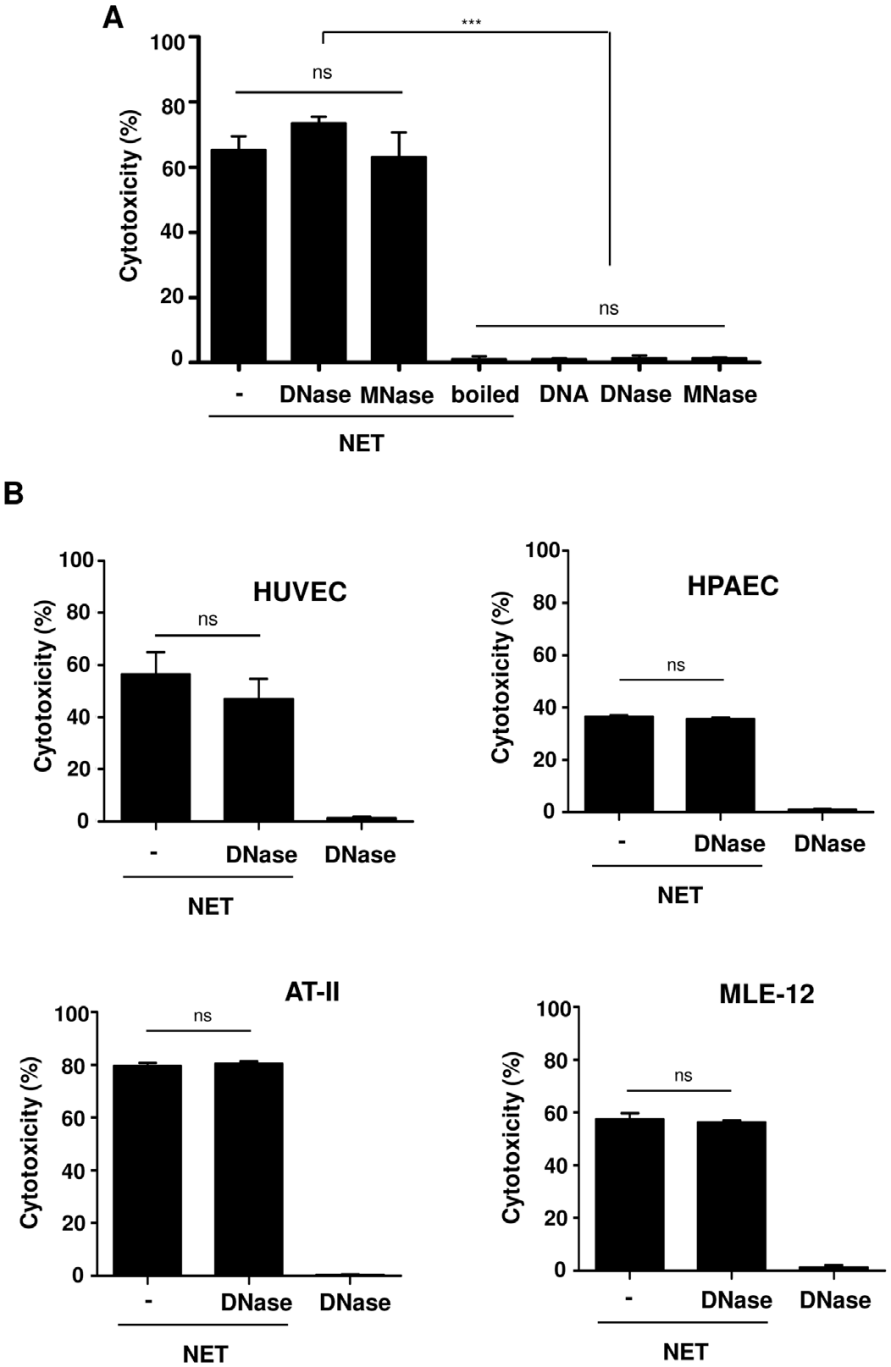
NET induce cytotoxicity in epithelial and endothelial cells independent of digestion. (A) The extent of cytotoxicity was measured after treatment of A549 cells for 16 h with undigested NET (−), completely (DNase), partially digested (MNase) or boiled forms of NET. The same concentration of DNA alone as DNA-NET (3.4 µg/ml) as well as DNase or MNase alone were used as controls. Shown are representative data of five independent experiments (mean 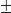 SD), ***p<0.001, and ns = non-significant. (B) The degree of cytotoxicity was measured after treatment of HUVEC, HPAEC, AT-II or MLE-12 cells for 16 h with undigested NET (−) or completely (DNase) forms of NET as well as DNA alone. Shown are representative data of three (except for AT-II, n = 2) independent experiments (mean 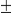 SD), ns = non-significant.

In order to investigate in detail the role of DNA digestion in NET-mediated cytotoxicity, different forms of NET were used: undigested (containing long DNA fibers), completely digested by DNase (containing mostly oligonucleotides) or partially digested by MNase (containing smaller DNA fragments but not nucleotides) (Fig. S1). Moreover, DNA alone, the same concentration as used in DNA-NET, was used as another control. The extent of cytotoxicity in A549 cells after incubation with NET substantially (approximately 60%) increased (Fig. 2A), and DNase or MNase treatment of NET did not change their cytotoxic activity. Incubation of cells with DNase, MNase or DNA alone as well as boiled NET did not provoke any appreciable cytotoxicity. To identify whether this effect of NET is also seen in other cells rather than A459, endothelial cells such as HUVEC and HPAEC as well as other lung epithelial cells such as MLE-12 and AT-II cells were incubated with undigested or DNA-digested NET, which induced similar cytotoxicity (Fig. 2B). Together, these data indicate that DNA digestion in NET does not change the cytotoxic activity of NET, and the DNA component in NET (either undigested or fragmented) is not responsible for its cytotoxic effect.

### Histones induce epithelial and endothelial cell death

**Figure 3:**
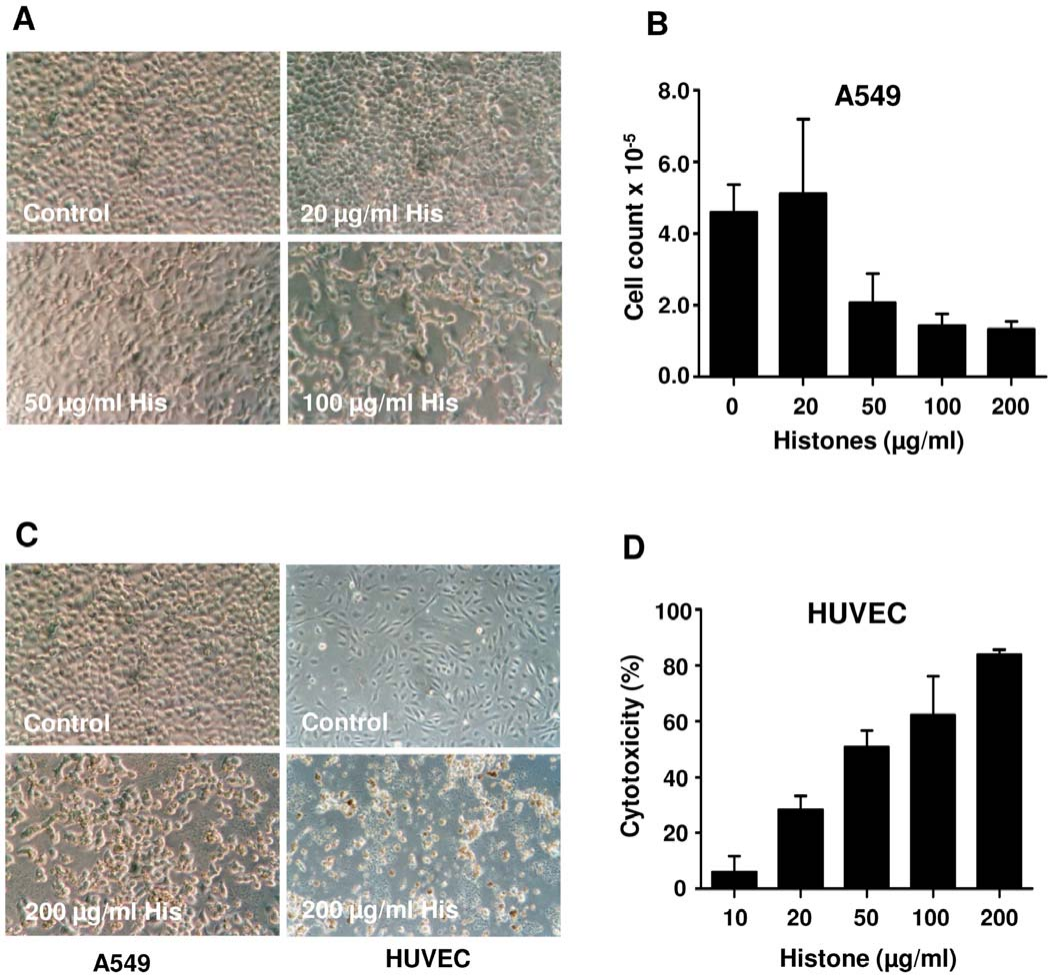
Histones induce epithelial and endothelial cell death. (A) A549 cells were treated for 16 h with different concentrations of histone type II-A, and the cell morphology was evaluated. (B) A549 cell numbers were counted after treatment with various concentrations of histones for 16 h. (C) HUVEC or A549 cells were treated with 200 µg/ml histones for 16 h or left untreated (control). (D) HUVEC were treated for 16 h with different concentrations of histones, and the extent of cytotoxity was measured. B and D are representative data of three independent experiments, and in A and C pictures are representative pictures from three independent experiments at 20× magnification.

Identification of spots from 2-D gel electrophoresis of NET by mass spectrometry confirmed the presence of different proteins, the majority of which had been described by Urban and co-workers [7]. Some additional newly identified proteins were listed as well (Table S2). To elucidate the relevance of histones as the major protein components of NET structure [7] for NET-mediated cytotoxicity, the influence of pure histones on both epithelial and epithelial cell death was investigated. NET contains all types of histones; therefore, commercially available purified histones (histone type-IIA, which includes all types of histones) were used for treatment with the cells. Incubation of epithelial and endothelial cells with histone type-IIA prevented cell growth, and provoked cytotoxicity in a concentration-dependent manner (Fig. 3). These data together with the previous studies [45] confirms the cytotoxic capability of histones on the host cells.

### Histone antibodies and polysialic acid decrease NET-mediated cytotoxicity

**Figure 4:**
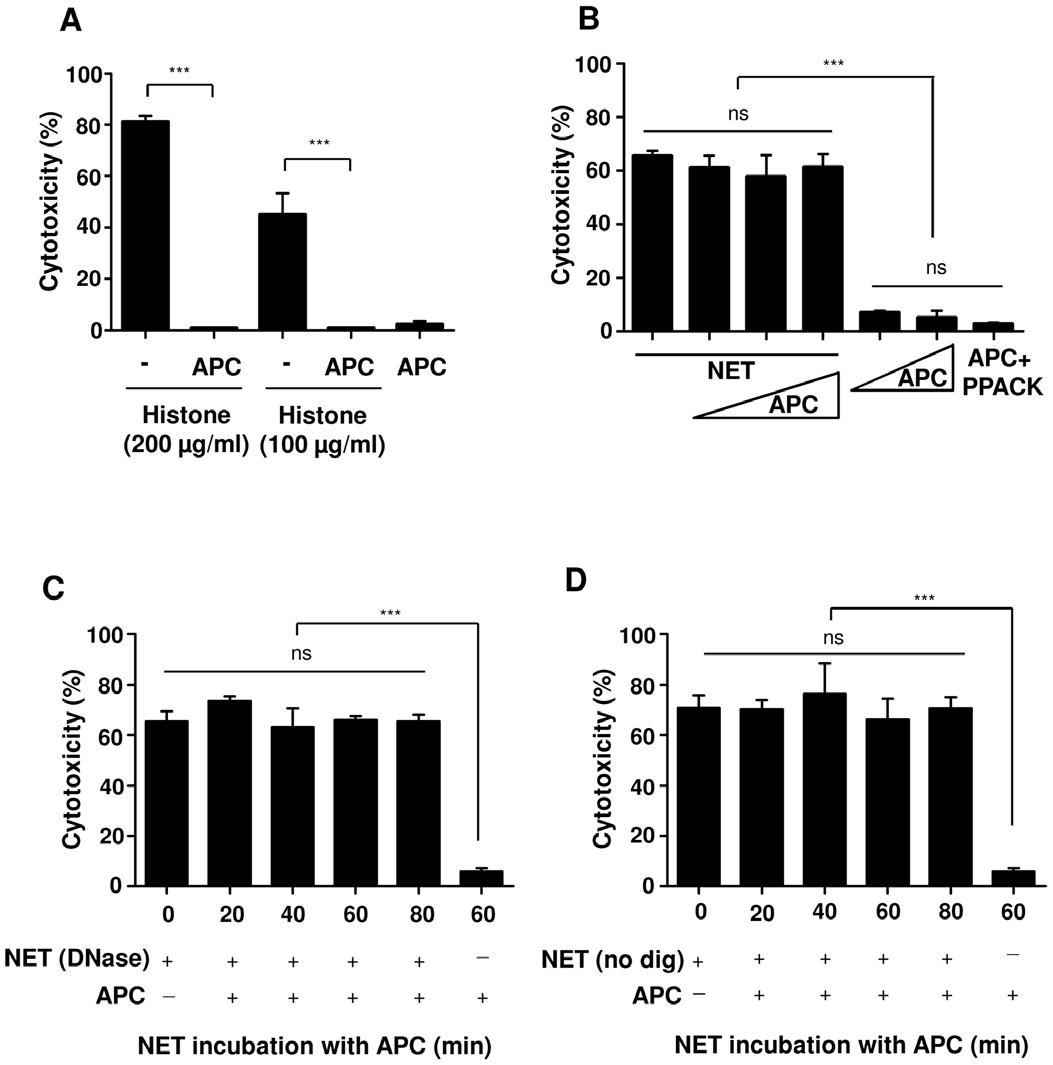
APC decreases epithelial cytotoxicity induced by histones but not by NET. (A) Histones (200 or 100 µg/ml), pre-incubated for 1 h at 37°C in the absence or presence of 100 nM human APC, were incubated with A549 cells for 16 h, followed by analysis of cytotoxicity. (B) NET were incubated with APC (mass ratio APC: NET proteins, 1∶5, 1∶2 and 1∶1) or without APC for 1 h at 37°C, followed by incubation with A549 cells for 16 h and measurement of cytotoxicty. APC alone or active-site blocked APC (APC+PPACK) were incubated with A549 cells for control. (C) DNase-digested and (D) undigested forms of NET were pre-incubated with 100 nM APC for 20 to 80 min before incubation with A549 cells for 16 h, followed by determination of cytotoxicty. Shown are representative data of four independent experiments (mean 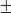 SD), ***p<0.001 and ns = non-significant.

**Figure 5:**
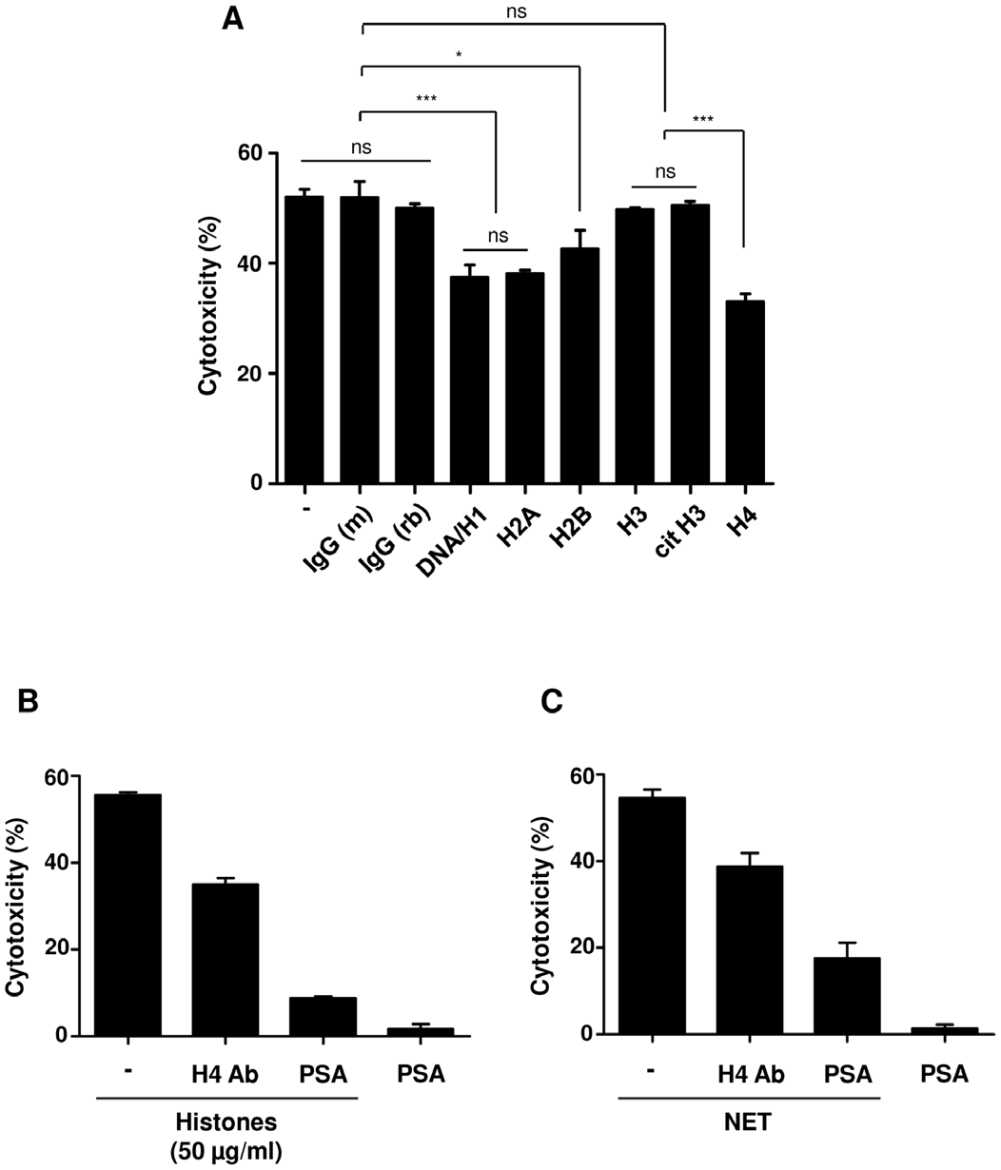
Histone antibodies and polysialic acid decrease NET-mediated cytotoxicity. (A) NET were pre-incubated with different antibodies against histones (DNA/H1, H2A, H2B, H3, citrullinated H3 [cit H3], H4) or with isotype-matched control antibodies. Antibody-treated NET or NET alone (−) were incubated with A549 cells for 16 h to analyze the cytotoxicity. Shown are representative data of three independent experiments (mean 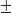 SD), ***p<0.001 and ns = non-significant. (B) Histones or (C) NET were pre-incubated with antibody against histone H4 or polysialic acid (PSA), followed by incubation with A459 cells for 16 h to analyze the cytotoxicity. Note that polysialic acid considerably decreased both histone- and NET-mediated cytotoxicity.

It was recently described that APC and histone antibodies can decrease histone-mediated cytotoxicity in endothelial cells [45]. Likewise, the cytotoxic activity of purified histones on A549 cells was significantly decreased by APC (Fig. 4A). In contrast, incubation of NET with APC even in high doses did not reduce NET-mediated cytotoxicity (Fig. 4B). Moreover, incubation of DNase or undigested forms of NET with APC up to 80 min could not decrease NET-mediated cytotoxicity (Fig. 4C and D). SDS-gel electrophoresis of NET proteins before or after incubation with APC and the subsequent identification with MALDI-TOF-MS did not show histone degradation (Fig. S2B). Likewise, two-dimensional difference in gel electrophoresis (2-D DIGE), which enables accurate analysis of differences in protein abundance between samples, did not show any difference between untreated NET and NET treated with APC (Fig. S2D), implying that histones in NET are protected against APC degradation. However, pre-incubation of NET with histone antibodies against DNA/H1, H2A, H2B and H4 but not H3 and citrullinated H3 significantly decreased NET-mediated cytotoxicity (Fig. 5A). Moreover, a recent study showed that polysialic acid, which is an extended and highly negatively charged glycan, can directly bind to histone and this interaction may be important for nervous system development and regeneration [46]. Pre-incubation of histones or NET with polysialic acid considerably reduced both histone- and NET-mediated cytotoxicity (Fig. 5B and C). These data indicate that the cytotoxicity of NET is in great part mediated by histones.

### Inhibition of neutrophil elastase does not inhibit the cytotoxic effect of NET

**Figure 6:**
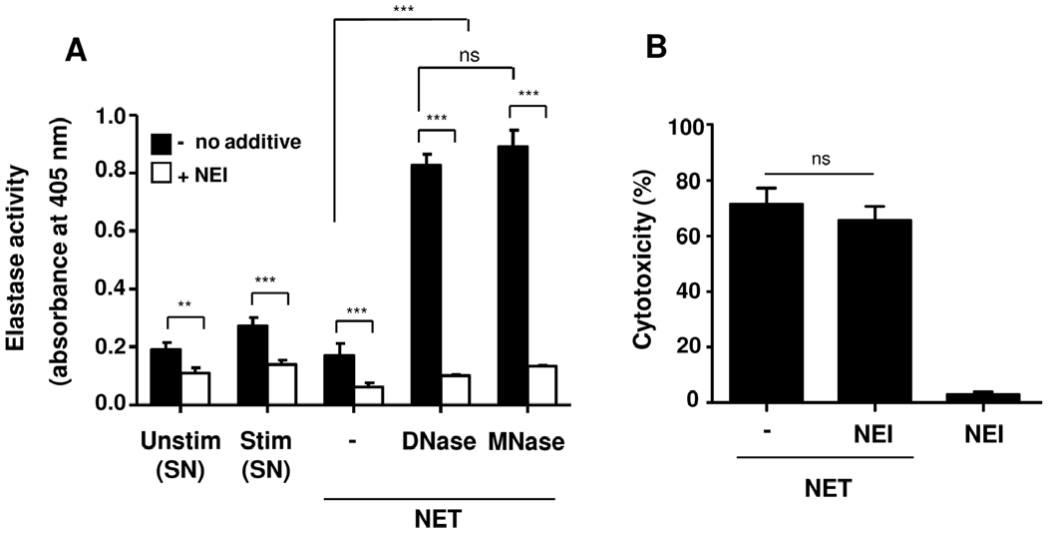
Inhibition of neutrophil elastase does not inhibit NET-induced cytotoxicity. (A) The supernatants of unstimulated (Unstim) or stimulated (Stim) neutrophils (50 nM PMA for 4 h) were collected and analyzed for elastase activity in the absence (*filled bars*) or presence (*open bars*) of neutrophil elastase inhibitor (NEI). Likewise, NET were isolated from stimulated cells and digested with DNase or MNase or kept undigested (−), followed by analysis of elastase activity in the same way. (B) Cytotoxicity of A549 cells was measured after 16 h treatment with NET (DNase-digested) in the absence or presence of NEI. Similar results were seen for MNase- or non-digested NET as well as with different NEI concentrations from 0.125 to 1 mM. Shown are representative data of three independent experiments (mean 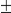 SD), ***p<0.001 and ns = non-significant.

Another abundant component of NET is neutrophil elastase [7]. To investigate the role of neutrophil elastase in NET-mediated cytotoxicity, activity of elastase in NET was measured before or after digestion. Increased activity of elastase in NET was observed after digestion of DNA either by DNase or MNase (Fig. 6A), and neutrophil elastase inhibitor significantly inhibited elastase activity in NET, indicating that neutrophil elastase is active in NET and its activity can be abolished by elastase inhibitor. However, using elastase inhibitor, even at high concentrations, could not reduce NET-mediated cytotoxicity in either digested or undigested NET (Fig. 6B).

### Inhibition of myeloperoxidase reduces NET-induced cytotoxicity

**Figure 7:**
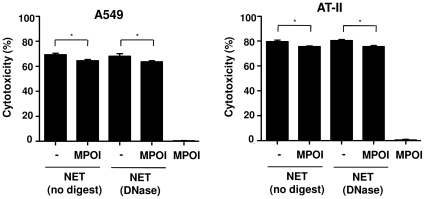
Myeloperoxidase inhibition moderately decreases NET-induced cytotoxicity of epithelial cells. Nondigested or DNase-digested NET were pre-incubated without or with myeloperoxidase inhibitor (MPOI), followed by incubation of NET with epithelial cells, A549 or AT-II cells, for 16 h and quantification of cytotoxicty. MPOI alone (37 ng/ml) was not toxic for the epithelial cells. Shown are representative data of three (for AT-II cells, n = 2) independent experiments (mean 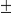 SD), *p<0.05 and ns = non-significant.

One of the other granular components of NET is myeloperoxidase (MPO) [5], [7]. MPO has an important role in defense against bacteria, viruses and fungi by conversion of hydrogen peroxidase to hypochlorous acid. However, MPO activity can also induce damage to adjacent tissue and, thus, contributes to the pathogenesis of different inflammatory diseases including pulmonary injury [47], [48]. Pre-incubation of MPO inhibitor with digested or non-digested NET led to reduction of NET-induced cytotoxicity in epithelial cells (Fig. 7). These data indicate that the cytoxicity of NET, besides histones, is partly mediated by MPO.

### NET formation in lung tissue and BALF of LPS-induced lung injury model

**Figure 8:**
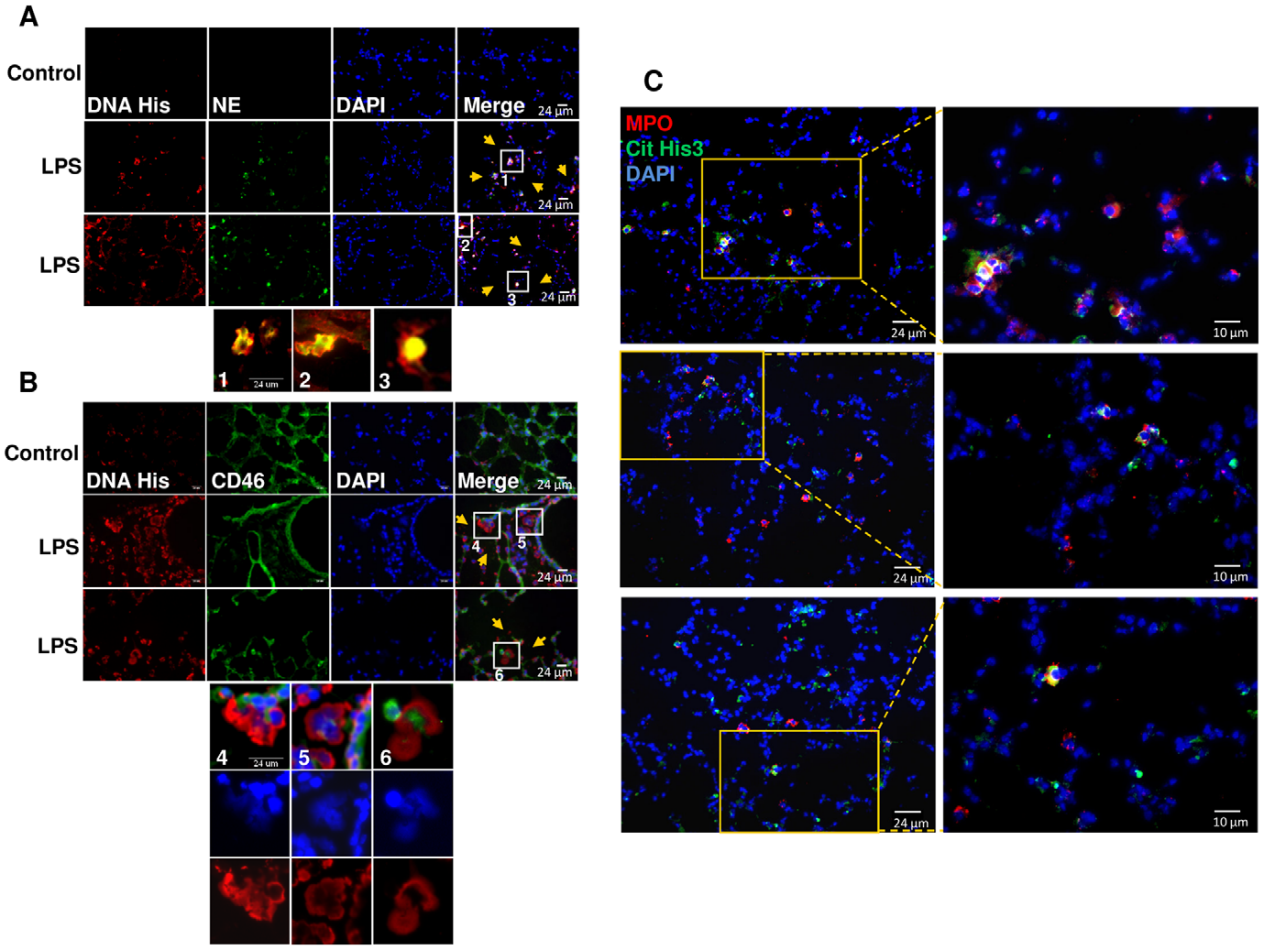
NET formation in LPS-induced lung injury mouse model. (A) Immunofluorescence staining of lung sections from mice after 24 h intratracheal LPS administration was performed, as compared to the control section, for DNA/histone (red), neutrophil elastase (green) and DAPI (blue). The higher magnification views of the insets (1, 2 and 3), which were randomly chosen, showed co-localization of neutrophil elastase (green) and DNA/histone (red) in NET structures. (B) Immunofluorescence staining of sections from PBS- or LPS-treated mice was performed for DNA/histone (red), CD46 (green) as a cell membrane marker and DAPI (blue). The randomly chosen insets (4, 5 and 6) showed NET formation in LPS-treated lungs in higher magnification views as appeared by extracellular chromatin, disintegration of the cell membranes as well as weak signal for DAPI (indication of chromatin decondensation). DAPI alone and DNA/histone alone were also shown for the insets 4, 5 and 6. In A and B, yellow arrows indicate some of the tissue destruction areas adjacent to NET. Shown are representative pictures of >10 fields of tissue staining. (C) Immunofluorescence staining of lung sections from mice after 24 h intratracheal LPS administration was performed for myeloperoxidase (MPO, red), citrullinated histone H3 (Cit His3, green) and DAPI (blue). The higher magnification views (right column) of the selected areas showed co-localization of myeloperoxidase with citrullinated histone H3 which indicate NET formation.

Under physiological conditions and in the absence of infectious or inflammatory stimuli, there are less than one million polymorphonuclear neutrophils (PMN) in the lung tissue and no PMN in the BALF [18]. To recruit PMN to the lung in considerable number, LPS was instilled intratracheally into mice to induce LPS-mediated acute lung injury [49]–[54]. NET formation was observed in mouse lung tissue after LPS treatment (as compared to the PBS controls), which was demonstrated by the co-localization of extracellular chromatin with neutrophil elastase (Fig. 8A) as well as the appearance of extracellular chromatin and disintegration of cell membranes (Fig. 8B). In general, decondensated chromatin in NET showed weak signal for DAPI staining, while it was detected strongly by DNA/histone antibody. CD46 receptor, which was used as a cell membrane marker, is present on all nucleated cells [55]–[57] and was used in this study to demonstrate the release of chromatin from the cells since NET formation requires the disintegration of the cell membrane. However, the non-frequent NET detected structures and the absence of long strands of chromatin in the tissue maybe due to the presence of short fragments of DNA-protein complexes in the airways, which is also confirmed recently by Douda and co-workers [58]. Several areas adjacent to NET structures showed tissue destruction which may be due to the cytotoxic effect of NET. Colocalizaion of myeloperoxidase with citrullinated histone H3, which is one of the histone modifications during NET formation [59], also indicated NET formation in lung tissue (Fig. 8C).

**Figure 9:**
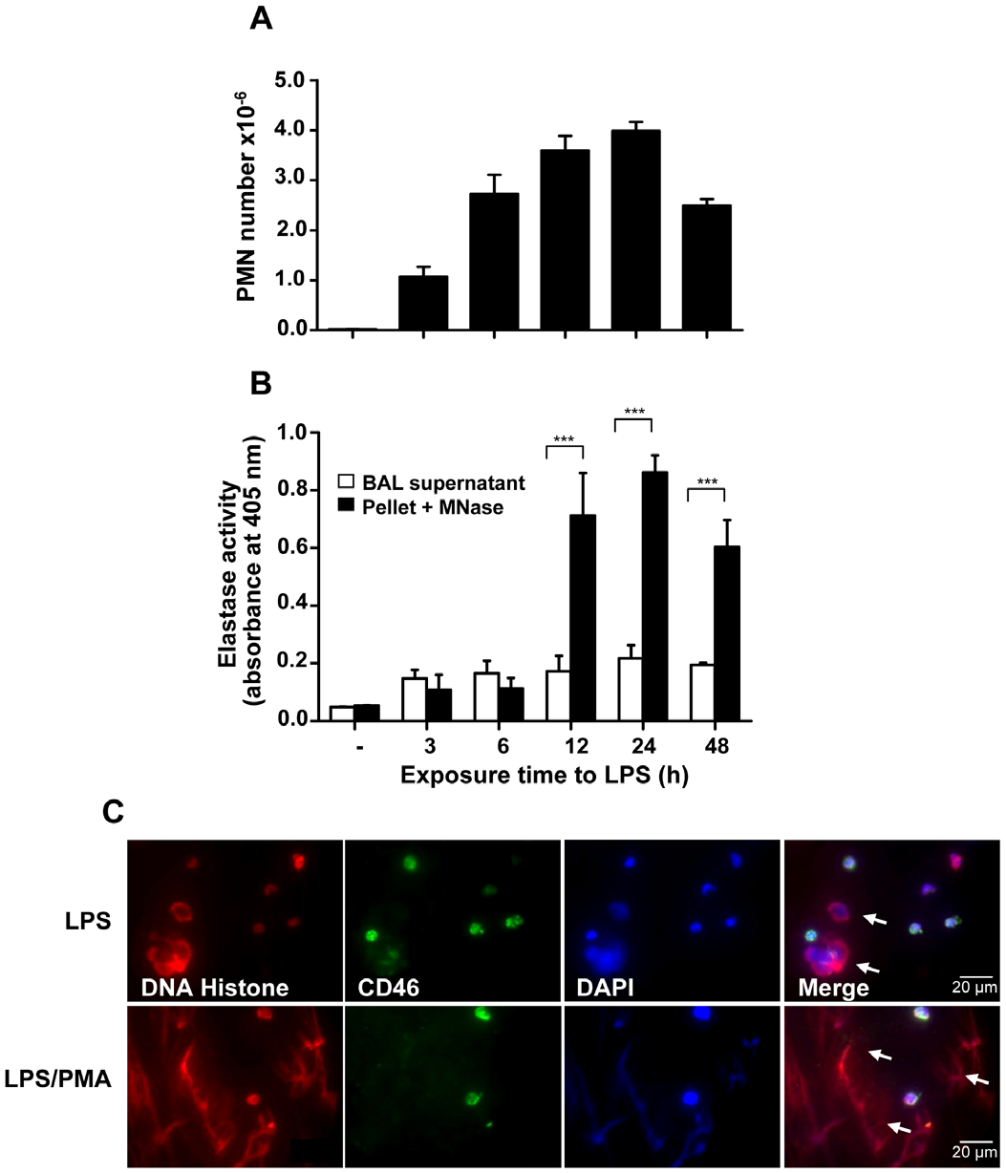
Neutrophils (PMN) in the BALF of mice produce NET after LPS treatment. (A) PMN, recruited to the BALF of mice following intratracheal LPS administration, were counted at different time intervals. (B) At the same time intervals, neutrophil elastase activity was measured in the supernatant of BALF (*free elastase, open bars*) as well as in the MNase-digested pellet of BALF (*NET-related elastase, filled bars*). (C) PMN were isolated from BALF of mice stimulated intratracheally with LPS for 24 h, and immunofluorescence staining of isolated cells was performed for DNA/histone (red), CD46 (green) and DAPI (blue) (*upper row*). Isolated PMN were further stimulated with PMA for 1.5 h (*lower row*); arrows indicate NET formation which is demonstrated by appearance of the extracellular chromatin, disintegration of the cell membranes as well as chromatin decondensation. Shown are representative data of three independent experiments (mean 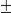 SD), ***p<0.001.

Moreover, after 3 to 48 h of stimulation with LPS, BALF was collected and the numbers of PMN were counted. Application of LPS provoked maximal recruitment of neutrophils after 24 h (Fig. 9A). Since treatment of NET with MNase leads to dissociation of NET-DNA from NET-proteins, neutrophil elastase activity in the BALF supernatant (free elastase) was compared to the activity of elastase in the NET structure after digestion with MNase (NET-related elastase). There was a significant increase in NET-related elastase activity after 12 h stimulation in comparison to the corresponding free elastase activity, indicating more NET formation after this time period of stimulation (Fig. 9B). Considerable increase in elastase activity in BALF after MNase digestion is in accordance with the data of Fig. 6A, where isolated NET showed increase in elastase activity after DNase or MNase digestion.

To characterize NET formation in BALF, mouse neutrophils in BALF after 24 h LPS stimulation were isolated with neutrophil-specific marker Ly-6G antibody, and stained for chromatin and cell membrane marker CD46. Since non-LPS stimulated neutrophils, as negative controls, could not be isolated from BALF [18], stimulation with PMA was used as a positive control. Therefore, some of these neutrophils were further stimulated with PMA, a potent inducer of NET formation, for 1.5 h *in vitro*. While LPS, which induces neutrophil recruitment, provoked NET formation (which was shown by the release of extracellular chromatin, disintegration of cell membrane as well as weak signal for DAPI) to a certain extent, it was more pronounced in cells stimulated with PMA (Fig. 9C). Together, these data demonstrate NET formation in the BALF and lung tissue in LPS-induced lung injury model.

## Discussion

Excessive NET formation or impairment of NET removal has been reported to be linked to chronic inflammatory diseases such as preeclampsia, small-vessel vasculitis and systemic lupus erythematosus [26], [28], [29], [31], [60], [61]. However, the direct influence of NET and their components on host cells have not been investigated. To verify that the cell destructive effect of neutrophils is merely due to NET components and not to other secreted components of neutrophils, the direct influence of isolated NET on alveolar epithelial cells (as the major cells responsible for gas exchange in the lung) as well as endothelial cells was examined in this study. Here, NET-induced cytotoxicity of epithelial and endothelial cells (whether primary or cell-line) in a dose-dependent manner was noted. Interestingly, DNA-digested NET (either completely digested or partially digested NET) did not change NET-mediated cytotoxicity. Moreover, boiled or denatured NET could not induce cytotoxicity which may further indicate that mostly proteins in NET are involved in NET-mediated cytotoxicity.

Bearing in mind that histones are the most abundant proteins of NET with bactericidal and leishmanicidal activity [5], [8], interference with histones by antibodies, polyanionic compounds or proteases should decrease NET-mediated cytotoxicity. Pre-treatment of NET with antibodies against different histones could decrease NET-mediated cytotoxicity to a varying degree; however, this treatment was not effective for all kinds of histones. Specific histone modification(s), unique structures of histones in complex with DNA and other proteins or histone degradation during NET formation or the protein complexes in NET could be responsible for such differences [7], [59], [62].

Using polyanionic compounds that mask or bind histones would be an alternative approach to interfere with histone-mediated cytotoxicity. Incubation of both isolated histones and NET with polysialic acid, which has been recently described as a binding partner for histone H1 [46], significantly reduced cytotoxicity. Yet, specificity of these compounds needs to be further explored.

Moreover, histones can be degraded with different proteases such as cytotoxic T lymphocyte protease granzyme A [63], activated nuclear proteasome [64] or APC. APC is a serine protease which plays an important role as natural anti-coagulant and also has been reported to degrade histones in an isolated system and thereby may reduce cell death in sepsis [45]. While our data showed that APC significantly decreased histones-mediated epithelial cytotoxicity in a purified system, incubation of NET (either nuclease-digested or not) with APC could not reduce NET-mediated cytotoxicity, although much higher concentrations of APC were used for treatment with NET as in a purified system. Analysis of NET compared to NET incubated with APC by SDS-gel electrophoresis and the subsequent protein identification did not show histone degradation after APC treatment (Fig. S2). Similarly, 2-D DIGE did not reveal any difference in the protein patterns between both samples. The observed protective effect could be due to (a) formation of DNA-histone complexes, (b) formation of complexes between histones and other proteins, (c) specific histone modification in NET [7], [59], (d) octamerization and decreased accessibility of histones in NET or (e) partial degradation of histones during NET formation [62]. The fact that histone-induced cytotoxicity in NET cannot be reduced by APC or completely abolished with histone antibodies still does not rule out the importance of these proteins in cytotoxicity, since APC and anti-histone antibodies may be the best option to decrease the cytotoxicity of histones in a purified system but not in NET.

Another abundant protein component of NET is neutrophil elastase [7], which has important roles in the clearance of invading pathogens. Moreover, elastase is able to mediate neutrophil-induced tissue damage and efficiently degrades extracellular matrix components [65]. Neutrophil elastase in NET showed increased activity after digestion of DNA. These data also support the presence of NET in BALF after LPS induction, as increased elastase activity was observed after DNA digestion. However, inhibition of neutrophil elastase in different forms of digested NET did reduce NET-mediated cytotoxicity. The presence of more cytotoxic components of NET (such as histones) may explain this effect such that inhibition of neutrophil elastase alone is not sufficient to suppress NET-mediated cytotoxicity. Alternatively, elastase in NET may induce cytotoxicity independent of its enzymatic activity; a possible mechanism that requires further attention.

MPO, another granular protein with anti-microbial activity, was also investigated for NET-mediated cytotoxicity in the present study. MPO has an important role in defense against bacteria by conversion of hydrogen peroxidase to hypochlorous acid. Nevertheless, MPO activity can also provoke damage to the adjacent tissues; therefore, it may contribute to the pathogenesis of several inflammatory diseases including pulmonary injury [47], [48]. It has been reported that MPO can provoke caspase-3 activation and apoptosis in HL-60 human leukemia cells [66]. Moreover, MPO can induce DNA strand breakage in lung epithelial cells [67]. Pre-incubation of NET with MPO inhibitor moderately decreased NET-mediated cytotoxicity of epithelial cells, indicating that MPO may also contribute to the cell-damaging capacity of NET.

NET is composed of DNA and different types of proteins with different sizes and cellular localization [7], and investigation of all of these proteins on NET-mediated cytotoxicity was beyond the scope of the present study. It should also be considered that different procedures for NET collection or various techniques for protein identification, such as gel-free mass spectrometry, may give rise to different results. So far, a standard procedure for NET collection, which truly confirms that the isolated NET contain all the materials and structures comparable to the *in vivo*-formed NET, has not been established. Therefore, it is essential to mention the exact procedure of NET collection in studies dealing with NET. Moreover, the diversity of NET proteins and their incomplete identification make the recognition and analysis of the cytotoxic parts of NET more complicated. Treatment of epithelial cells with NET induced up-regulation of proteins with different kinds of functions from regulation of cell cycle to glycolysis and proteasome degradation pathway. Interestingly, several of these proteins are known to be up-regulated during cell death and apoptosis (Table S1) which further supported our data about NET-mediated cytotoxicty.

NET structures and areas with tissue destruction adjacent to NET were observed in the LPS-treated mice as well as in other studies during influenza pneumonitis [68]. The absence of long strands of chromatin in the tissue, which could be due to the presence of short fragments of DNA-protein complexes in the airways [58], may complicate the analysis of NET in lung tissue. It is also worthwhile to consider that not only NET components but also secretion of granular proteins can lead to tissue destruction. During the preparation of the present study, Narasaraju and co-workers showed that co-incubation of neutrophils with HUVEC resulted in endothelial cell death [68]. However, in the present study, epithelial and endothelial cells were incubated with isolated NET and not with neutrophils to exclude the effect of other components of neutrophils such as secreted enzymes and proteins which are not bound to NET.

Lung epithelial cell death is not only observed in ALI but also in other diseases such as cystic fibrosis. Progressive inflammation and infection results in destruction of airways in the lungs of cystic fibrosis patients, and patients' lungs contain extracellular DNA which accumulates in the airway due to the chronic bacterial infection [21]. Using immunocytochemistry, we observed clear structural similarity between isolated NET and the supernatant of cystic fibrosis sputum where chromatin fibers were decorated with granular proteins such as elastase (data not shown). This leads us to propose that NET may be responsible for cytotoxic effects in the broncho-alveolar space of cystic fibrosis patients. These observations were supported by a very recent study [69]. Although aerosolic therapy with recombinant human DNase has been linked with an improvement in airflow obstruction and a decrease in the number of infectious respiratory exacerbations in some cystic fibrosis patients, this is not true for all cases [70]. As reported in this study, digestion of DNA in NET was not sufficient to abolish NET cytotoxicity, and this could be one reason that DNase therapy is not successful in all cystic fibrosis patients.

In this study, we tested the direct effect of NET on epithelial and endothelial cells and found a case for histones in cytotoxicity. So far, any study which estimates the exact amount of DNA-derived NET in humans suffering from acute lung injury has not been documented. Nevertheless, the DNA-NET concentrations used in this study are in the range of DNA concentration in the sputum of the cystic fibrosis patients [71] as well as plasma DNA concentration of systemic lupus erythematosus patients [72]. Other studies also showed that the extracellular DNA in these patients is predominantly derived from NET [29]–[31], [69].

However, the *in vivo* situation is far more complex. Phagocytosing cells remove dead cells and their debris (perhaps NET components too). Furthermore, the ciliary motion in the lung prevents the maintenance of dead cells in one place for a longer period of time. It has to be further investigated whether one of the reasons which promote lung destruction in patients with ALI or cystic fibrosis is the impairment of NET removal mechanisms. In addition to lung cells, NET showed cytotoxic effect on other cells, such as HUVEC (also skin and neuronal cells, data not shown), which may refer to the general cytotoxic capability of NET.

## Supporting Information

Figure S1 **Agarose gel electrophoresis of neutrophil- and NET-derived DNA after treatment with MNase or DNase.** Isolated NET or lysed unstimulated neutrophils were kept undigested or treated with DNase or MNase, followed by separation of DNA on 1.5% agarose gel.
(TIF)

Figure S2 **APC degrades isolated histones but not NET-associated proteins.** (A) SDS-gel electrophoresis (15%) of histones and (B) NET proteins before or after incubation with 100 nM APC were carried out, and several bands were identified with MALDI-TOF-MS. In (A) 10 times higher amount of APC was loaded as a control. (C) 2-D gel electrophoresis (pH 7–11) of NET (left) and NET treated with APC (right) were performed. (D) NET (Cy3, green) and APC-treated NET (Cy5, red) labeled proteins were separated by 2-D gel electrophoresis. Gels from both samples were overlaid with a minor space in the vertical orientation (left gel) or exactly superimposed (right gel). No difference in the gel profiles was noted.
(TIF)

Table S1 **Identified up-regulated proteins in A549 cells after incubation with NET.** A549 cells were treated with NET, and the up-regulated proteins were identified by MALDI-TOF-MS. A wide range of proteins with different functions and cellular localizations were up-regulated. Roles in cell death for several proteins (indicated by ^a^) have been reported based on www.uniprot.org.
(DOC)

Table S2 **Identified NET proteins by 2-D gel electrophoresis and MALDI-TOF-MS.** 2-D gel electrophoresis was performed for NET-proteins with isoelectric focusing at different pH ranges (3–10 and 7–11). From 32 randomly chosen spots in NET, we identified 13 different proteins from which 9 proteins have been already described by Urban and co-workers [7]. Four different proteins were identified for the first time (indicated by ^a^). Several functions of these proteins have been mentioned based on www.uniprot.org.
(DOC)

Materials and Methods S1 (DOC)
