# Supplementary figures and images for: Neutrophil Extracellular Traps Directly Induce Epithelial and Endothelial Cell Death: A Predominant Role of Histones
Source: PLoS One. 2012 Feb 28;7(2):e32366. doi: 10.1371/journal.pone.0032366 (PMC3289648; doi:10.1371/journal.pone.0032366)

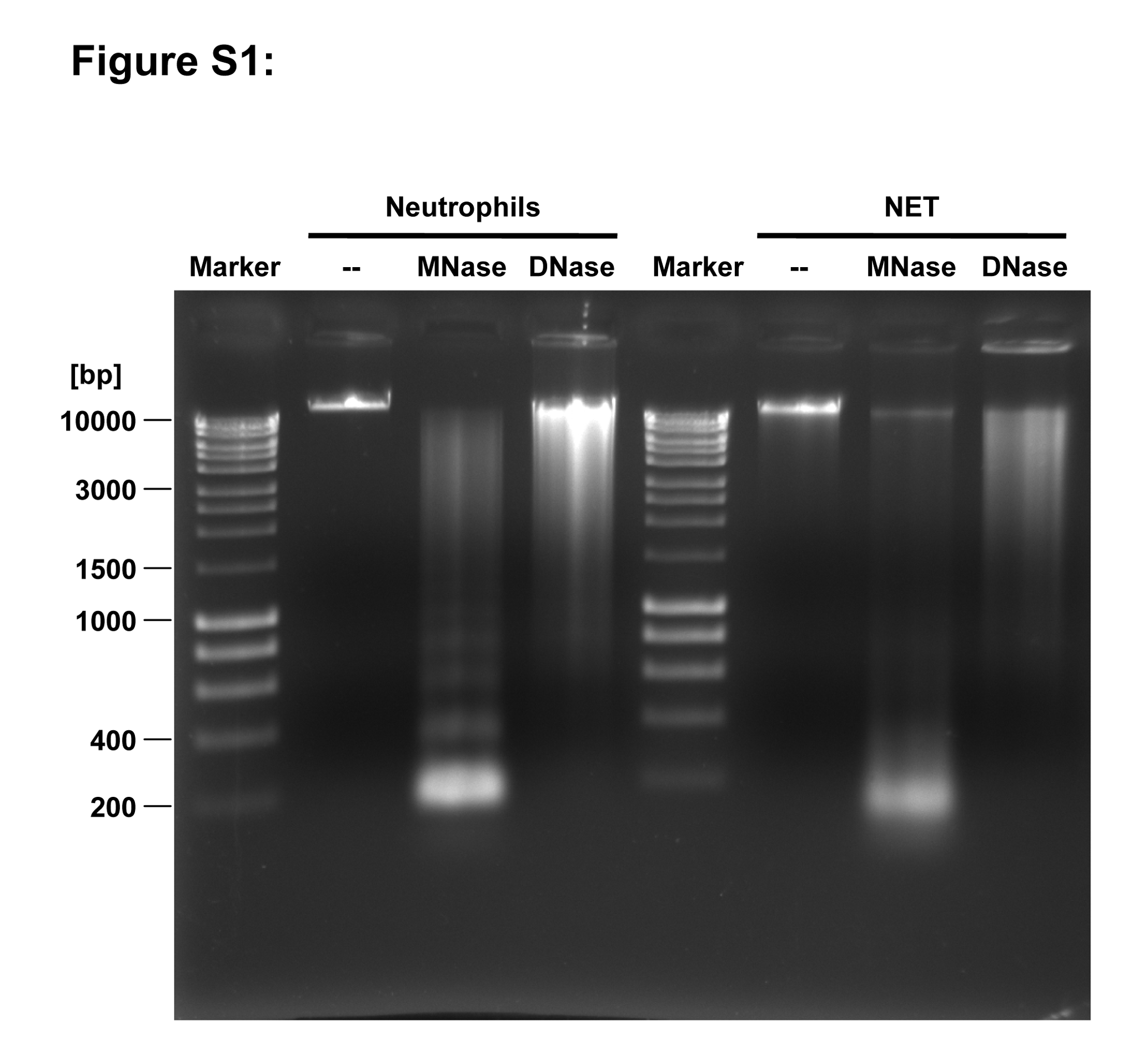

Supplement: Figure S1 — Agarose gel electrophoresis of neutrophil- and NET-derived DNA after treatment with MNase or DNase. Isolated NET or lysed unstimulated neutrophils were kept undigested or treated with DNase or MNase, followed by separation of DNA on 1.5% agarose gel. (TIF) [file pone.0032366.s001.tif]

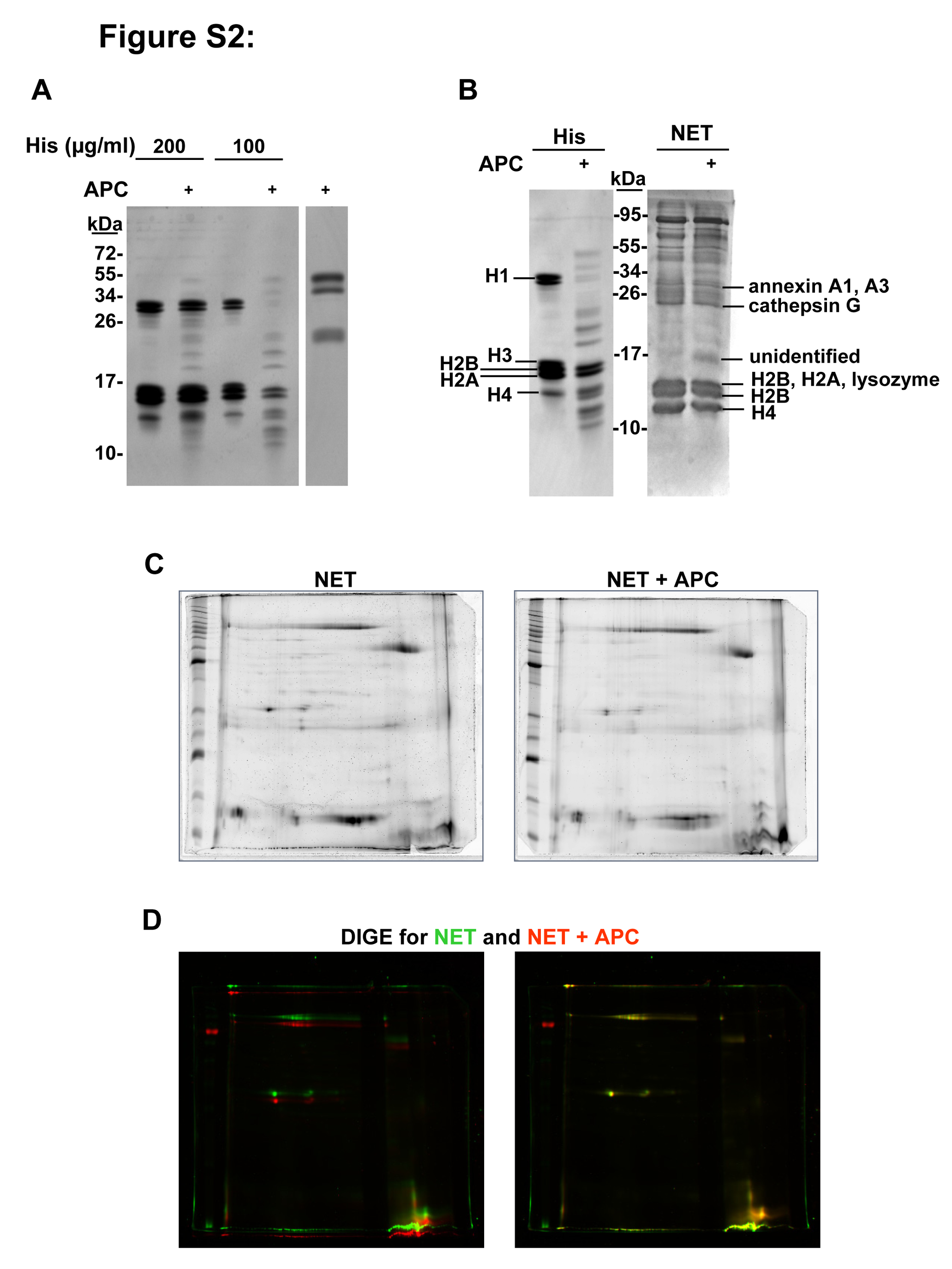

Supplement: Figure S2 — APC degrades isolated histones but not NET-associated proteins. (A) SDS-gel electrophoresis (15%) of histones and (B) NET proteins before or after incubation with 100 nM APC were carried out, and several bands were identified with MALDI-TOF-MS. In (A) 10 times higher amount of APC was loaded as a control. (C) 2-D gel electrophoresis (pH 7–11) of NET (left) and NET treated with APC (right) were performed. (D) NET (Cy3, green) and APC-treated NET (Cy5, red) labeled proteins were separated by 2-D gel electrophoresis. Gels from both samples were overlaid with a minor space in the vertical orientation (left gel) or exactly superimposed (right gel). No difference in the gel profiles was noted. (TIF) [file pone.0032366.s002.tif]
