# Supplementary material for: Neutrophil Extracellular Traps Directly Induce Epithelial and Endothelial Cell Death: A Predominant Role of Histones
Source: PLoS One. 2012 Feb 28;7(2):e32366. doi: 10.1371/journal.pone.0032366 (PMC3289648; doi:10.1371/journal.pone.0032366)
Supplement: Table S1 — Identified up-regulated proteins in A549 cells after incubation with NET. A549 cells were treated with NET, and the up-regulated proteins were identified by MALDI-TOF-MS. A wide range of proteins with different functions and cellular localizations were up-regulated. Roles in cell death for several proteins (indicated by a) have been reported based on www.uniprot.org. (DOC) [file pone.0032366.s003.doc]

**Table S1.** **Identified up-regulated proteins in A549 cells after incubation with NET**

| **Identified proteins** | **Cellular component** |
| --- | --- |
| Elongation factor 1-beta | Cytosol |
| Heterogeneous nuclear ribonucleoprotein C-like 1 | Nucleus |
| Isoform C2 of Heterogeneous nuclear ribonucleoproteins C1/C2 | Nucleus  Spliceosome |
| Isoform 2 of Heterogeneous nuclear ribonucleoprotein A1 | Cytoplasm  Nucleus Spliceosome |
| Heterogeneous nuclear ribonucleoprotein K | Cytoplasm Nucleus Spliceosome |
| Proliferating cell nuclear antigen | Nucleus |
| Isoform 2 and 1 of Tropomyosin alpha-3 and 4 chain | Cytoplasm  Cytoskeleton |
| Aldo-keto reductase family 1 member C3 | Cytoplasm |
| Annexin A1 | Cell membrane Cell projection Cilium Cytoplasm Membrane Nucleus |
| Annexin A2 | Basement membrane Extracellular matrix Secreted |
| Glyceraldehyde-3-phosphate dehydrogenase ***^a^*** | Cytoplasm Membrane Nucleus |
| Protein disulfide-isomerase | Cell membrane Endoplasmic reticulum Membrane |
| Prohibitin ***^a^*** | Membrane Mitochondrion Mitochondrion inner membrane |
| Ubiquitin carboxyl-terminal hydrolase isozyme L1 | Cytoplasm |
| F-actin-capping protein subunit alpha-1 | Cytoplasm Cytoskeleton |
| Aldo-keto reductase family 1 member B10 | Cytoplasm |
| Transaldolase | Cytoplasm |
| Glutathione S-transferase omega-1 | Cytoplasm |
| Protein DJ-1 (Oncogene DJ1, Parkinson disease protein 7) ***^a^*** | Cytoplasm  Mitochondrion  Nucleus |

| **Identified proteins** | **Cellular component** |
| --- | --- |
| Retinal dehydrogenase 1 | Cytoplasm |
| Isoform 1 of Triosephosphate isomerase | Cytosol |
| Nitrilase homolog 2 | Cytoplasm |
| Poly (rC)-binding protein 1 | Cytoplasm Nucleus |
| Stress-induced-phosphoprotein 1 | Cytoplasm Nucleus |
| Peptidyl-prolyl cis-trans isomerase A | Cytoplasm |
| Superoxide dismutase [Mn], mitochondrial ***^a^*** | Mitochondrion |
| Vitamin K epoxide reductase complex subunit 1-like protein 1 | Membrane |
| Isoform 1 of Mitochondrial import receptor subunit TOM40 homolog | Membrane Mitochondrion Mitochondrion outer membrane |
| Phosphoglycerate kinase 1 | Cytoplasm |
| Isoform 1 of Phosphoserine aminotransferase | Cytoplasm |
| Profilin-1 | Cytoplasm Cytoskeleton |
| 60 kDa heat shock protein, mitochondrial ***^a^*** | Mitochondrion |
| Heat shock protein beta-1 ***^a^*** | Cytoplasm  Cytoskeleton  Nucleus |
| Proteasome subunit beta type-2 ***^a^*** | Cytoplasm Nucleus Proteasome |
| Proteasome subunit beta type-3 ***^a^*** | Cytoplasm Nucleus Proteasome |
| Proteasome subunit beta type-4 ***^a^*** | Cytoplasm Nucleus Proteasome |
| Isoform 1 of Proteasome activator complex subunit 3 ***^a^*** | Cytoplasm Nucleus Proteasome |
| proteasome 26S non-ATPase subunit 13 isoform 2 ***^a^*** | Proteasome |

***a*** Refers to the proteins which are involved in cell death based on www.uniprot.org
