# Supplementary material for: Neutrophil Extracellular Traps Directly Induce Epithelial and Endothelial Cell Death: A Predominant Role of Histones
Source: PLoS One. 2012 Feb 28;7(2):e32366. doi: 10.1371/journal.pone.0032366 (PMC3289648; doi:10.1371/journal.pone.0032366)
Supplement: Table S2 — Identified NET proteins by 2-D gel electrophoresis and MALDI-TOF-MS. 2-D gel electrophoresis was performed for NET-proteins with isoelectric focusing at different pH ranges (3–10 and 7–11). From 32 randomly chosen spots in NET, we identified 13 different proteins from which 9 proteins have been already described by Urban and co-workers [7]. Four different proteins were identified for the first time (indicated by a). Several functions of these proteins have been mentioned based on www.uniprot.org. (DOC) [file pone.0032366.s004.doc]

**Table S2. Identified NET proteins by 2-D gel electrophoresis and MALDI-TOF-MS**

| **NET-proteins** | **Functions *^b^*** |
| --- | --- |
| Annexin A1 ***^a^*** | Membrane fusion, Exocytosis, Calcium/phospholipid-binding protein |
| Lactoferrin | Cellular iron ion homeostasis, Iron ion transport, Antimicrobial activity |
| Profilin-1 ***^a^*** | Actin cytoskeleton organization, Platelet activation |
| Alpha-enolase | Glycolysis, Plasminogen activation,  Transcription regulation |
| Protein S100-A8 and A9 | Calcium-binding protein, Chemotaxis, Antimicrobial activity |
| Glyceraldehyde 3-phosphate dehydrogenase ***^a^*** | Glycolysis, Nitrosylase activities, Transcription, RNA transport, DNA replication, Apoptosis |
| Actin, cytoplamsmic 1 and 2 | Adherens junction organization, Cytoskeleton, Cell motility |
| Leukocyte elastase inhibitor (Serpin B1) ***^a^*** | Regulation of proteolysis |
| Plastin-2 | Actin-binding protein, T cell activation |
| Myeloperoxidase | Microbicidal activity |
| Proteinase 3 | Serine protease which degrades extracellular matrix |

***a*** Refers to the NET-associated proteins which are identified in this study for the first time.

***b*** Refers to the described functions of the proteins based on [www.uniprot.org](http://www.uniprot.org/).
