# Supplementary material for: Neutrophil Extracellular Traps Directly Induce Epithelial and Endothelial Cell Death: A Predominant Role of Histones
Source: PLoS One. 2012 Feb 28;7(2):e32366. doi: 10.1371/journal.pone.0032366 (PMC3289648; doi:10.1371/journal.pone.0032366)
Supplement: Materials and Methods S1 — (DOC) [file pone.0032366.s005.doc]

**Supplementary material and methods**

**Two-dimensional (2-D) gel electrophoresis:** 2-D gel electrophoresis was performed for NET samples or NET-treated epithelial cells. For proteomic analysis of epithelial cells after NET treatment, confluent A549 cells in 24-well plates were treated with 800 μl non-digested or DNase-digested NET for 1, 3, 6 or 8 h, and untreated cells were used as controls. After incubation, cell supernatants were discarded, wells were washed once with PBS and the cells were collected with lysis buffer for 2-D gel electrophoresis. For proteomic analysis of NET proteins, NET samples were treatment with 10 U/ml DNAse I and 50 µl protease inhibitor for mammalian cells (Sigma-Aldrich) for 2 h at 37°C. Thereafter, the samples were precipitated with acetone and redissolved in TRIS-buffer (10 mM TRIS, 5 mM CaCl_2_, 5 mM MgCl_2_; pH 8.0). Protein extraction was performed by ultrasonication in 100 μl 6 M urea (Sigma-Aldrich), 2 M thiourea (Sigma-Aldrich), 4% CHAPS (Roth, Germany), 1% DL-Dithiothreitol (DTT) (Fluka, Germany) and 2% Pharmalyte 3-10 (GE Healthcare). Protein quantification was performed with 2-D Quant kit (GE Healthcare). IPG-strips (pH 3-10 for epithelial proteins, pH 3-10 and pH 7-11 for NET-proteins, all non linear) were rehydrated at 20°C with the protein extract. On each strip 400 µg protein were applied and isoelectric focusing was performed with 32.05 kVh. Thereafter, the IPG-strips were equilibrated for 10 min in 2 ml equilibration stock solution (ESS; 6 M urea 0.1 mM EDTA, 0.01 % bromphenol blue, 50 mM Tris-HCl pH 6.8, 30 % glycerol, v/v), 15 min in 2 ml ESS I (10 ml ESS containing 200 mg SDS, 100 mg DTT) followed by 15 min in ESS II (10 ml ESS containing 200 mg SDS, 480 mg iodacetamide). Protein separation in the second dimension was performed by electrophoresis on 12.5% SDS polyacrylamide gels according to Laemmli [S1]. Electrophoresis was carried out in a Hoefer 600 system with the following program: 15 min at 15 mA/gel and 5 h at 110 mA at 25°C. Gels were stained with coomassie brilliant blue R-250 (Serva, Germany) and scanned with a GS-800 densitometer (BioRAD, Germany).

**Tryptic in-gel digestion of proteins**: Spots of interest (the spots which showed up-regulation in all NET-treated epithelial cells in comparison to the untreated cells by student t-test, 98% confidence) were excised with the ExQuest spot cutter (BioRAD) and proteins were digested with trypsin on a liquid handling roboter system (MicroStarlet, HamiltonRobotics).

**Matrix-assisted laser-desorption ionization time-of-flight mass spectrometry (MALDI-TOF-MS):** MALDI-TOF-MS was performed on an Ultraflex TOF/TOF mass spectrometer (Bruker Daltonics, Germany) equipped with a nitrogen laser and a LIFT-MS/MS facility. The instrument was operated in the positive-ion reflectron mode using 2.5-dihydroxybenzoic acid (Sigma) and methylendiphosphonic acid (Fluka) as matrix. Sum spectra consisting of 200–400 single spectra were acquired. For data processing and instrument control the Compass 1.1 software package consisting of FlexControl 2.4, FlexAnalysis 3.0 and BioTools 3.0 was used.

**Database search**: Proteins were identified by MASCOT peptide mass fingerprint search (http://www.matrixscience.com) using the human IPI database. For the search a mass tolerance of 75 ppm was allowed and carbamidomethylation of cysteine as global modification and oxidation of methionin as variable modification were used.

**Differential in gel electrophoresis** (**DIGE) derivatization and 2-D gel electrophoresis:** For DIGE derivatization using the minimal labeling kit, 100 µg of NET-proteins before or after treatment with APC were derivatized with Cy3 and Cy5, respectively, according to the instructions of the manufacturer (GE Healthcare). From both samples 50 µg proteins were mixed and labeled with Cy2 as an internal standard. All three samples were mixed and subjected to 2-D gel electrophoresis and proteins were separated according to their pI by isoelectric focusing on IPG strips (13 cm, pI 7-11 nl, GE Healthcare) with 32.05 kVh. For the second dimension, 12.5% polyacrylamide gels were used. Gels were scanned with a VersaDoc system from BioRAD. Gel images were analyzed with the PdQuest software (BioRAD).

**Analysis of neutrophil- and NET-derived DNA by agarose gel electrophoresis**

NET was collected as it explained in NET production, isolation and quantification and either kept non-digested or were treated with MNase or DNase. As a control, unstimulated neutrophils were mixed with passive lysis buffer (Promega; Germany), followed by treatment with DNase or MNase, or it was kept undigested. Thereafter, each preparation was purified with phenol chloroform extraction and precipitated with 99% ethanol. The resulting pellet was redissolved in double distilled water and subjected to agarose gel elecrophoresis on 1.5% agarose gel together with an appropriate marker.

**Supplementary reference**

S1. Laemmli UK (1970) Cleavage of structural proteins during the assembly of the head of bacteriophage T4. Nature 227: 680-685.
